# Emotion Regulation and Physiological Reactivity in the Parent-Child Relationship: A Preliminary Study of an Online Attachment-Based Program for Parents of Preadolescents with Behavioral Disorders

**DOI:** 10.1192/j.eurpsy.2024.341

**Published:** 2024-08-27

**Authors:** M. Tironi, S. Charpentier Mora, F. Bizzi

**Affiliations:** ^1^University of Genoa, Genoa, Italy

## Abstract

**Introduction:**

Behavioral disorders have been defined as a “health crisis” of modern times that has a significant impact on the parent-child relationship. In this scenario, the emotional regulation (ER) of each partner plays a central role and serves a protective factor, configuring as an area to intervene. The Connect Parent Group, an attachment-based intervention for parents, has shown evidence of effectiveness. However, its online version (e-Connect) has not yet garnered specific evidence related to emotional and physiological regulation in parents and preadolescents.

**Objectives:**

This study aimed to explore changes in the short and medium term regarding ER abilities - both self-reported and measured through physiological indices - in parents and preadolescents with behavioral disorders, building upon initial findings from an online parenting intervention.

**Methods:**

28 parents (82.1% mothers, 17.9% fathers, M_age = 47.48, SD = 4.73) and their 28 preadolescents with behavioral disorders (M_age = 11.22 years, SD = 2.69, 35.7% girls) were recruited from child neuropsychiatry services in Northern Italy and subsequently took part in the pilot study. They were assessed at three time points: before intervention (T1), one months after the intervention (T2) and at 6-months follow-up (T3). ER were assessed with a multimethod approach: parents and children completed a self-report questionnaire (i.e., Difficulties in Emotion Regulation Scale and How I Feel, respectively) and then they interact during a stress-task in which physiological parameters (i.e., Galvanic Skin Response, GSR; Heart Rate/Beat per Minute, BPM) have been measured.

**Results:**

Regarding self-reported ER, mixed-effects regression models showed an improvement in parent emotion dysregulation between T1 and T3 (*p*=0.004), a decrease in preadolescents’ negative emotions (*p*=.012) between T1 and T2 and a lower emotion intensity in preadolescents between the three-time points (*p*=.003). Regarding physiological ER, the two overall models of GSR and BPM were not significant for both parents and children. Yet GSR correlations within three-time points were positive and significant for children (T1-T2: *r*=.58; T1-T3: *r*=.68) but not for parents, while BPM correlations between T1 and T2 were significant for parents (*r*=.49) but not for children.

**Conclusions:**

The online attachment-based parenting program appears to have contributed to a reduction in emotional dysregulation in parents and preadolescents, which seems to persist to some extent in the medium term. The non-significant results at the physiological level may suggest that changes reported by parents and children through self-report questionnaires do not align with changes in the physiological response to interpersonal stress experienced after an online intervention. Clinical and research implications will be discussed.

**Disclosure of Interest:**

None Declared

